# Access to Services Within the Entrusted Budgets in Primary Healthcare in Poland from 2022 to 2025

**DOI:** 10.3390/healthcare13182358

**Published:** 2025-09-19

**Authors:** Magdalena Mrożek-Gąsiorowska

**Affiliations:** Health Economics and Social Security Department, Institute of Public Health, Faculty of Health Sciences, Jagiellonian University Medical College, 8 Skawinska Street, 31-066 Krakow, Poland; magdalena.mrozek-gasiorowska@uj.edu.pl

**Keywords:** primary health care, health inequities, access to primary care, health services accessibility, coordinated care, integrated care, Poland

## Abstract

**Background**: Entrusted budgets were introduced as part of the primary healthcare (PHC) system in Poland in July 2022. This initiative aimed to increase the role of PHC and enhance the accessibility of diagnostic services and specialist consultations/advice for patients. **Methods**: Data from the National Health Fund (NHF) databases regarding contracts between the NHF and healthcare providers in the field of PHC from 2022 to 2025 were analyzed. The share of contracts with entrusted budgets in the total number of PHC physician contracts was estimated in individual voivodships, as well as in counties, using the example of the Małopolskie Voivodship. It was assessed whether there were significant differences between voivodships and counties as well as the pace of implementation of the new solution. **Results**: Only 43.1% of PHC physicians have signed contracts with the NHF for coordinated care services for 2025, with this percentage varying significantly between voivodships, ranging from 24.8% in Opolskie Voivodship to 66.3% in Lubelskie Voivodship (*p* < 0.0001). For the vast majority of voivodships, no statistically significant increase in the share of service providers was demonstrated in the period from 2022 to 2025. Access to services between counties is also highly varied (from 10.0% to 76.5%), although the differences were not statistically significant (*p* = 0.217). **Conclusions**: The results indicate regional and local inequalities in access to services. It is necessary to implement incentive mechanisms within the contracting of health services between the NHF and providers that will ensure equal access to PHC services within entrusted budgets for all patients. The range of available services should be equal regardless of at which PHC facility a patient is registered. The current regulations concerning entrusted budgets, including the voluntary involvement of service providers, are not sufficient.

## 1. Introduction

Access to diagnostic services and specialist outpatient care (AOS) in Poland has been difficult for many years, and the waiting time for a specialist visit often spans several months [1,2]. The COVID-19 pandemic further restricted access to healthcare services. During the COVID-19 pandemic in 2020–2021, the number of consultations with a specialist in AOS decreased by approximately 20% compared to previous years. There was also a deterioration in access to healthcare services in primary healthcare. The number of the most frequently provided primary health care consultations, i.e., physician outpatient consultations (face-to-face or teleconsultations), was increasing in the pre-pandemic period but in 2020 it declined by nearly 18% and continued in the following year. Delays in treatment and interruptions in the provision of healthcare services caused by the COVID-19 pandemic have led to the emergence of a significant “health debt” in the population [3].

All primary healthcare services are provided free of charge to patients in Poland. The patient chooses a primary healthcare physician and is added to their list if the physician has available capacity. Capitation method is the main mechanism to finance primary healthcare by the public payer in Poland, along with a fee-for-services method used to pay for specific services and lump sum payments to finance night and holiday care. Poland began implementing various new public funding mechanisms for healthcare services in 2022 to improve this situation and reduce the accumulated health needs. These include entrusted budgets and motivational bonuses in primary healthcare (PHC). This paper presents an evaluation of the entrusted budgets (diagnostic entrusted budget and coordinated care entrusted budget) implemented in PHC.

Various changes to healthcare provider payment methods have been implemented both in response to the COVID-19 pandemic [4] and before and after it [5,6] in many countries. The aim of these changes is to adapt the healthcare system in a given country to the changing reality, on the one hand, thereby reducing the growth of medical care expenditure, and on the other hand, expand the scope of services and improve its quality [7]. One of the main recommendations is that the changes introduced should be subject to periodic evaluation, particularly with regard to access to and quality of services. However, in many cases, the evaluation of the reforms are scarce [4,5]. When shaping reforms, it is also necessary to take into account the expected barriers and prepare appropriate interventions that will help to efficiently implement the planned reforms [8].

The need for changes in primary healthcare financing was highlighted also by The Lancet Global Health Commission in 2022 [9]. Reforms of the primary healthcare system have been carried out in recent years, among others, in Poland, the Czech Republic, Hungary, and Slovakia. The gradual and voluntary implementation of the entrusted budgets in Poland was in line with the World Bank recommendations and was intended to enable less-ready clinics to learn from the first implementers [6,10].

In this study, the availability of healthcare services included in the scope of entrusted budgets within primary healthcare from 2022 to 2025 in Poland was assessed. The aim of the study was to answer the questions whether the number of service providers offering coordinated care has increased significantly since the introduction of this mechanism for financing primary healthcare services and whether there are significant differences between voivodships and individual counties within a voivodship in relation to the participation of service providers in the implementation of health care services within the entrusted budgets. The analysis of data from all years since the introduction of this funding mechanism allows for the evaluation of the pace of implementation of entrusted budgets and addresses the question of whether it is sufficient to achieve the expected performance indicators set in 2022. The recommendations resulting from this research may help improve access to services within the entrusted budgets for all patients. The results of the analysis can also be the lesson from implementation experience and may help other countries avoid the mistakes made during the implementation of the new coordinated care system within PHC in Poland.

## 2. Materials and Methods

### 2.1. Data Sources

The analysis focuses on data from the National Health Fund (NHF) database regarding contracts signed with healthcare providers for the implementation of primary healthcare services from 2022 to 2025.

The analysis used the following data available in the NHF databases:

- Informator o zawartych umowach [Information on signed contracts] [11];
- Baza Aktów Własnych NFZ [The database of the acts of the NHF] [12];
- Przychodnie POZ realizujące opiekę koordynowaną [Primary care clinics providing coordinated care] [13].

### 2.2. Data Selection and Analysis

The “Informator o zawartych umowach” database of the NHF is updated regularly by the public payer. It provides data on the scope of individual contracts between the NHF and healthcare providers in all service areas. However, it does not include aggregated data. For this study, data from 2022–2025 were extracted, as the first entrusted budget in primary healthcare was introduced in mid-2022. The contracts were extracted from the NHF database in which the following services were contracted:

1. Świadczenia lekarza POZ [Primary healthcare physician services] (code: 01.0010.094.01);
2. Budżet powierzony diagnostyczny [Diagnostic entrusted budget] (code: 01.0010.119.11);
3. Budżet powierzony opieki koordynowanej [Coordinated care entrusted budget] (code: 01.0010.120.11).

This NHF database allows for the retrieval of data on the number of healthcare providers who signed a contract with a regional NHF branch. In the case of analyzing data at the county level, healthcare providers were assigned to a given county based on the address of their headquarters. All healthcare providers were assigned to a specific category (no healthcare provider records were rejected from the analysis). In a few cases, the healthcare provider’s headquarters was located outside the Małopolskie Voivodship. In these cases, the location of service provision was verified based on data from the clinic’s website, and these entities were assigned to the county where the branch was located or, if applicable, where the largest number of branches in the Małopolskie Voivodship were situated.

Based on this data, the number of contracts in which an entrusted budget was contracted was calculated as a percentage of the total number of primary healthcare physician contracts. Data were extracted in January 2025 and the most recent data update was conducted on 5 March 2025 for the data regarding the year 2025.

In the analysis, the “Przychodnie POZ realizujące opiekę koordynowaną” database of the NHF was also used. The database only presents data on facilities providing coordinated care in individual voivodships, counties and locations. It does not allow for estimating the involvement of physicians in providing coordinated care services. Additionally, for a given healthcare provider, the database may present several records depending on the number of service provision addresses. However, it does contain data on the scope of coordinated care services provided by healthcare providers who decided to provide services, as selected by them (diabetology, endocrinology, cardiology, pulmonology, nephrology). Currently, this database contains information as of 1 February 2025.

The analysis also includes current legal regulations regarding the conditions for concluding and implementing contracts related to primary healthcare in Poland based on the “Baza Aktów Własnych” database of the NHF.

### 2.3. Statistical Methods and Software

Data were analyzed using MS Excel. The chi-square test was used to assess whether the differences observed between the voivodships and counties in the percentage of providers who decided to provide services within the entrusted budgets were statistically significant. A linear trend (linear regression) analyses, using the least-squares method were also performed to assess whether the increase in the share of services providers in the implementation of entrusted budgets from 2022 to 2025 is statistically significant.

## 3. Results

### 3.1. Assessment of the Implementation Process and Scope of the Entrusted Budgets in Primary Healthcare

Only selected and usually relatively inexpensive diagnostic tests, such as blood count and other hematological tests, biochemical and immunochemical serum tests, urine and stool tests, some other diagnostic tests and images, are financed within the capitation rate within primary health care in Poland (Table 1) [14].

In July 2022, additional diagnostic tests were introduced in Poland, available within the diagnostic entrusted budget of primary healthcare and free of charge for the patient. This budget covered, for example, an anti-CCP test and an *H. pylori* antigen stool test [15,16] (Table 1). These tests were available earlier only within specialist outpatient care or, in some cases, hospitalization.

As of 1 October 2022, the range of healthcare services financed within the primary healthcare physician’s entrusted budget was expanded. A second entrusted budget was introduced, coordinated care entrusted budget, which allocates additional funds to ensure coordination of care for the patient. Primary care physicians have been given the opportunity to order a range of new tests, as well as to utilize other services not previously available, such as consultations with specialist doctors. Under this budget, additional laboratory and imaging diagnostic tests, as well as advice and consultations with specialists in the area of cardiology, diabetology, pulmonology, allergology, endocrinology and nephrology, comprehensive advice and other advice (including diet and educational advice) for patients with suspected or already diagnosed selected chronic diseases (diabetes, hypertension, ischemic heart disease and other heart diseases, thyroid diseases, and chronic lower respiratory diseases, and later also chronic kidney failure) were to be financed [16,17]. The goal of this budget was to allow for more effective detection of the most common chronic diseases and facilitate comprehensive patient care without the need to refer them to a specialist providing services as part of outpatient care.

Further changes were introduced in the following years. The immunoglobulin level tests and HIV screening test were introduced to the scope of the diagnostic entrusted budget on 3 November 2023 and on 2 May 2025, respectively. The pricing of individual services has also been adjusted since the introduction of the entrusted budgets [16].

The current scope and funding level for individual healthcare services are presented in Table 1.

**Table 1:** Diagnostic healthcare services available within the capitation rate and entrusted budgets in primary healthcare in Poland.

Types of Health Services	Healthcare Services	Date of Introduction	Current Service Cost Financed by the NHF [PLN] [18]
Diagnostic services financed under the capitation rate	(1) Hematological tests, including the following: Peripheral blood count with platelets, Peripheral blood count with differential and platelets, Reticulocytes, Erythrocyte sedimentation rate (ESR) (2) Biochemical and immunochemical tests in blood serum, including the following: Sodium, Potassium, Ionized calcium, Iron, Iron—Total Iron Binding Capacity (TIBC), Transferrin concentration, Glycated hemoglobin (HbA1c) concentration, Urea, Creatinine, Glucose, Oral glucose tolerance test, Total protein, Protein electrophoresis, Albumin, C-reactive protein (CRP), Uric acid, Total cholesterol, HDL cholesterol, LDL cholesterol, Triglycerides (TG), Total bilirubin, Direct bilirubin, Alkaline phosphatase (ALP), Aspartate aminotransferase (AST), Alanine aminotransferase (ALT), Gamma-glutamyltransferase (GGTP), Amylase, Creatine kinase (CK), Total acid phosphatase (ACP), Rheumatoid factor (RF), Antistreptolysin O (ASO) titer, Thyroid-stimulating hormone (TSH), Hepatitis B surface antigen (HBsAg), VDRL, Free triiodothyronine (FT3), Free thyroxine (FT4), PSA—Prostate-specific antigen, total, Total calcium concentration (3) Urine tests, including the following: General urine test with evaluation of physical and chemical properties and microscopic sediment examination, Quantitative protein measurement, Quantitative glucose measurement, Quantitative calcium measurement, Quantitative amylase measurement (4) Stool tests, including the following: General examination, Parasitic examination, Occult blood test—immunochemical method (5) Coagulation tests, including: Prothrombin time (INR), Activated partial thromboplastin time (APTT), Fibrinogen (6) Microbiological tests, including the following: Urine culture with antibiogram, Throat swab culture with antibiogram, Stool culture for Salmonella and Shigella, SARS-CoV-2 antigen test using the Vaccination Distribution System (SDS) (7) Electrocardiographic (ECG) test at rest (8) Ultrasound tests, including the following: Thyroid and parathyroid ultrasound, Salivary gland ultrasound, Kidney, ureter, and bladder ultrasound, Abdominal and retroperitoneal ultrasound, including preliminary prostate gland assessment, Peripheral lymph node ultrasound (9) Radiological images, including the following: Chest X-ray in AP and lateral projection, Bone X-rays: (a) spine (whole spine) in AP and lateral projection, (b) spine (segmental) in AP and lateral projection, (c) limbs in AP and lateral projection, (d) pelvis in AP and lateral projection, Skull X-ray, Sinus X-ray, Abdominal X-ray (overview)	Not applicable	Not applicable
Diagnostic entrusted budget	(1) Ferritin	1 July 2022	24.15
	(2) Vitamin B12		25.36
	(3) Folic acid		19.32
	(4) Anti-CCP		68.84
	(5) CRP—rapid quantitative test (children up to 6 years of age)		16.91
	(6) Anti-HCV antibodies		46.20
	(7) *H. pylori* antigen in stool—cassette test		27.77
	(8) *H. pylori* antigen in stool—laboratory test		73.67
	(9) Strep-test		14.49
	(10) Total Immunoglobulin E (IgE)	3 November 2023	28.35
	(11) Specific Immunoglobulin E (IgE) with a 10-point panel for respiratory allergies		117.87
	(12) Specific Immunoglobulin E (IgE) with a 10-point panel for food allergies		117.87
	(13) Specific Immunoglobulin E (IgE) with a 20-point panel for respiratory allergies		198.27
	(14) Specific Immunoglobulin E (IgE) with a 20-point panel for food allergies		198.27
	(15) HIV screening test (anti-HIV antibodies and p24 antigen)	2 May 2025	41.79
Coordinated care entrusted budget	(1) BNP (NT-pro-BNP)	1 October 2022	112.07
	(2) Albuminuria (albumin concentration in urine)		9.48
	(3) UACR (urine albumin/creatinine ratio)		37.42
	(4) Anti-TPO (antibodies against thyroid peroxidase)		48.88
	(5) Anti-TSHR (antibodies against TSH receptors)		81.76
	(6) Anti-TG (antibodies against thyroglobulin)		42.76
	(7) Stress ECG (exercise ECG test)		199.70
	(8) 24 h Holter ECG (24-h ECG recording)		165.52
	(9) 48 h Holter ECG (48-h ECG recording)		185.75
	(10) 72 h Holter ECG (72-h ECG recording)		216.93
	(11) 24 h Holter BP (24-h blood pressure recording)		165.52
	(12) Doppler ultrasound of carotid arteries		128.32
	(13) Doppler ultrasound of the veins of both lower limbs #		193.23
	(14) Doppler ultrasound of the arteries of both lower limbs #		193.23
	(15) Transthoracic echocardiogram (ECHO)		194.93
	(16) Spirometry		67.92
	(17) Spirometry with bronchodilator test		120.20
	(18) Targeted fine-needle aspiration biopsy of the thyroid (up to 2 procedures, for adults)		481.67
	(19) Targeted fine-needle aspiration biopsy of the thyroid (at least 3 procedures, for adults)		967.04
	(20) Consultation with a specialist doctor (a primary care physician—a specialist in a selected field)		98.61
	(21) Advice—specialist doctor and patient (a patient and a specialist in a selected field)		159.78
	(22) Educational advice		55.57
	(23) Diet advice		66.79
	(24) Comprehensive advice—Individual Care Plan		227.48

#—until July 2023, reported together as Doppler ultrasound of lower limb vessels.

The addition of entrusted budgets into the catalogue of services available within primary healthcare was intended to improve accessibility, quality, and efficiency of care, as well as patient satisfaction. The introduction of coordinated care was meant to be a major step toward improving the healthcare situation, strengthening the role of primary care physicians, and increasing patients’ chances of a quick diagnosis.

Entrusted budgets introduced in Poland since July 2022 are additionally financed by the public payer on a “fee-for-service” basis, meaning payment is made for the healthcare services provided, up to the limit of the entrusted budget specified in the contract between the NHF and the healthcare provider. The amount of the budget is initially determined based on the population size and age structure of the patients on the primary healthcare physician’s list and also the estimated average number of services covered by the budget, and in subsequent settlement periods, it is adjusted taking into account factors such as the number of services performed and reported in the previous settlement period. In the case of the coordinated care entrusted budget, specialists listed in the schedule are also taken into account. A primary healthcare physician providing care to a patient within the scope of services covered by the coordinated care entrusted budget is required to conduct at least one comprehensive advice session per calendar year. The National Health Fund finances no more than 9 dietary or educational advice sessions in total per calendar year, as well as one comprehensive advice session. Additionally, the total value of consultations with a specialist (primary care physician—specialist) cannot exceed 3% of the coordinated care entrusted budget in the settlement period. It was also assumed that the initial consultation is carried out within the capitation rate of the primary care physician, with necessary diagnostic tests financed within this rate. Additional diagnostic tests for the diagnosis or exclusion of a chronic disease may be billed from the coordinated care entrusted budget if they are ordered during the initial consultation. However, if a chronic disease is not confirmed, the comprehensive advice cannot be billed. Service providers can deliver all or selected services from those indicated in the entrusted budget for coordinated care. Coordinated care services are provided in at least one of the following areas:

- Diagnosis and treatment of hypertension, heart failure, chronic ischemic heart disease, and atrial fibrillation;
- Diagnosis and treatment of prediabetic conditions or diabetes;
- Diagnosis and treatment of bronchial asthma and chronic obstructive pulmonary disease;
- Diagnosis and treatment of hyperthyroidism, diagnosis and treatment of hypothyroidism, as well as diagnosis of single and multiple thyroid nodules;
- Diagnosis and treatment of chronic kidney disease [16].

The provision of services within the entrusted budget by healthcare providers is voluntary. A service provider who wants to deliver coordinated care services must submit an appropriate application to the National Health Fund to extend the contract to include the new scope of services. The new scope has expanded the tasks of the care coordinator. Therefore, service providers interested in delivering services within the entrusted budget for coordinated care, who do not have a contract for the scope “care coordination—coordinator’s tasks”, should also submit an application for the scope “care coordination—coordinator’s tasks” [16].

### 3.2. Access to Services Within the Entrusted Budgets in Primary Healthcare

Taking into account that the provision of services within the entrusted budget by primary healthcare providers is voluntary, not all providers have chosen to offer these services.

Almost all primary healthcare providers decided to offer services within the diagnostic entrusted budget introduced in July 2022 during its first year of operation. Currently, this range of additional diagnostic services is widely available to all patients within primary healthcare.

In the case of the coordinated care entrusted budget, which was introduced slightly later, in October 2022, the pace of implementing these services has been decidedly slower from the beginning. Table 2 presents data on the number of service providers delivering primary healthcare physician services, as well as the number and percentage of these service providers offering services within the coordinated care entrusted budget from 2022 to 2025 in Poland. A significant increase in the number of service providers offering coordinated care within the entrusted budget between 2022 and 2025 was demonstrated only in the case of the Podkarpackie Voivodship (*p* = 0.049) (Table 2).

In 2022, only between 0.5% and 4.6% of primary healthcare physician contracts, depending on the voivodship, included the coordinated care entrusted budget. Nearly one in three service providers decided to sign a contract with the National Health Fund in this area in the first full year after the introduction of these healthcare services into the guaranteed benefits catalogue in primary healthcare. Even at this stage, significant differences between voivodships can be observed. In the following year, the pace at which service providers engaged in delivering services within the coordinated care entrusted budget slowed down, and by 2024, a level of 40.2% was achieved nationwide. Data for 2025 show that additional service providers are not opting to provide coordinated care services. The share of primary healthcare physician contracts with the coordinated care entrusted budget increased by only a few percentage points in 2025 compared to 2024. Currently, 43.1% of primary healthcare physician contracts include the coordinated care entrusted budget, but regional and local disparities remain large.

**Figure 1:**
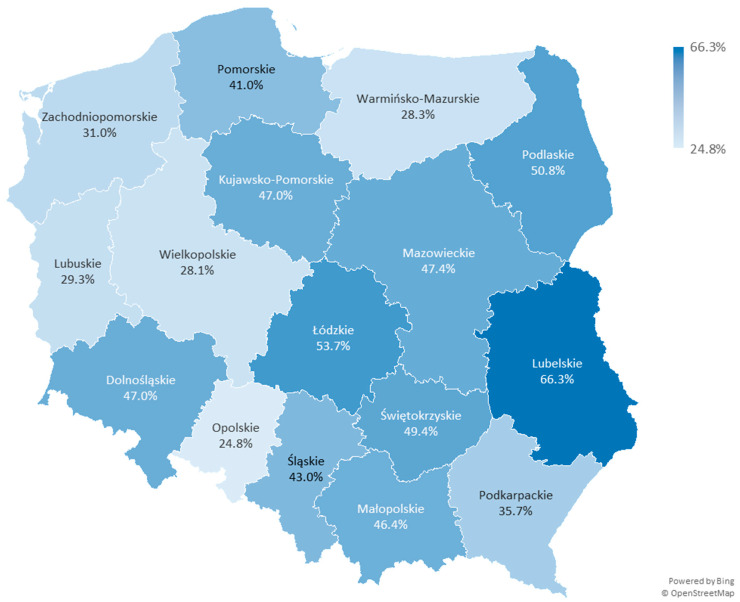
Percentage of service providers delivering services within the coordinated care entrusted budget in each voivodship in 2025.

The situation is the best in the Lubelskie Voivodship, where two out of three (66.3%) healthcare providers who contracted primary healthcare physician services offer services within the coordinated care entrusted budget. On the other hand, fewer than one in three providers offer these services in the Opolskie, Wielkopolskie, Warmińsko-Mazurskim, Lubuskie, and Zachodniopomorskie Vouvodships (Figure 1). The analysis demonstrated that the proportion of providers with entrusted budgets differed significantly across voivodship (χ^2^ = 271.7, df = 15, *p* < 0.0001).

**Figure 2:**
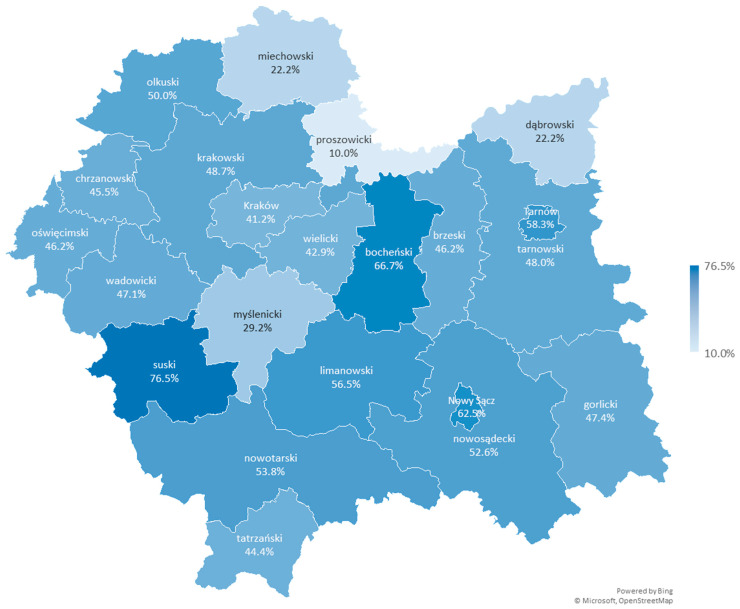
Percentage of service providers delivering services within the coordinated care entrusted budget in the counties of the Małopolskie Voivodship in 2025.

The Małopolskie Voivodship overall achieves a rate close to the national average (46.4% of contracts in 2025). However, a more in-depth analysis of this voivodship shows that local differences between counties are even greater than those observed when comparing individual voivodships (Table 3 and Figure 2). The range of the results varies from 10% to 76.5%, although the analysis did not show significant differences between individual counties (χ^2^ = 25.72, df = 21, *p* = 0.217).

In the Proszowicki County, which borders the Kraków County and Krakowski County, only one out of ten providers (10%) decided to offer coordinated care. This provider (Samodzielny Publiczny Zespół Opieki Zdrowotnej w Proszowicach) is registered in the county’s headquarters, in the town of Proszowice. Two other providers, also based in Proszowice, have not yet chosen to offer coordinated care. Similarly, all other providers registered in smaller towns and villages located in the Proszowicki County, such as Bobin, Klimontów, Koszyce, Kościelec, Nowe Brzesko, Pałecznica, and Radziemice, have not opted to provide coordinated care either. A similar situation occurs in the neighboring or nearby Miechowski County and Dąbrowski County, where only 22.2% of providers are currently offering the evaluated services.

On the other hand, in the Suski County, 76.5% of primary healthcare providers (13 out of 17 entities) decided to contract coordinated care entrusted budget. This county borders Slovakia and is located far from the voivodship capital of Kraków, without direct borders with either Kraków County or Krakowski County. Among the providers offering coordinated care in this county, there are entities registered both in the county’s main town, Sucha Beskidzka, and in smaller towns and villages such as Bystra-Sidzina, Maków Podhalański, and Zawoja. Only some of the providers registered in locality like Budzów, Jordanów, and Stryszawa offer coordinated care (more than one entity is registered in these locality). Additionally, one provider registered in Zembrzyce, which directly borders Sucha Beskidzka, also does not offer these services.

The opinions so far have indicated that the program is characterized by higher accessibility in urbanized areas. The analysis of data at the county level does not clearly indicate that a higher percentage of healthcare providers delivering coordinated care is found in larger urban centers. The rate for Kraków, a city with county rights and the capital of a Małopolskie Voivodship, is lower than the regional average and lower than in the surrounding counties (Krakowski County and Wielicki County), where the rates are slightly higher than in Kraków itself. In the case of other larger towns that have city-county status, such as Tarnów and Nowy Sącz, a rate about 10% higher than in the surrounding counties (Tarnowski County and Nowosądecki County, respectively) can be observed.

Among the healthcare providers delivering coordinated care, only 35.7% have decided to offer the full range of coordinated care services nationwide. Accordingly, 82.8%, 69.0%, 86.6%, 71.5%, and 43.5% of healthcare providers offering coordinated care have chosen the scope of diabetology, endocrinology, cardiology, pulmonology, and nephrology, respectively. The percentages for the Małopolskie Voivodship are 79.6%, 69.1%, 82.9%, 68.1%, and 34.2%, respectively. Considering that more than half of the healthcare providers do not offer coordinated care services at all, patient access to coordinated care services is much lower than estimated when accounting for the presence of any scope of this care in the contracts between the NHF and healthcare providers. It also depends on the chronic disease the patient has.

## 4. Discussion

The aim of the study was to assess access to healthcare services within the entrusted budgets in primary healthcare. The article offers an in-depth examination of the rollout and availability of entrusted budgets in Poland’s primary healthcare system between 2022 and 2025. It sheds light on the complexities and developments related to the incorporation of budgetary tools, particularly those focused on diagnostics and coordinated care in Poland. Based on the analysis of data for the years 2022–2025, it was shown that access to these services is not universal. More than half of the service providers do not provide coordinated care at all. Those who have decided to provide these services often offer them only in selected areas. Moreover, the increase in the participation of service providers in delivering services has slowed down. Additional service providers are not deciding to deliver coordinated care. The weakness of the coordinated care program in primary care turned out to be that primary care clinics are not obliged to join it within a given period of the program implementation, so not all patients will benefit from the reform.

Reforms of healthcare service financing methods encounter various types of barriers. Among the barriers observed in various countries are stakeholder opposition, design-related difficulties, shortcomings in implementation mechanisms, resource limitations, market challenges, legal impediments, informational gaps, and negative public perception [8,19]. The pace of implementation of the entrusted budgets as a common solution in primary healthcare in Poland is insufficient. The results obtained can be linked to the Diffusion of Innovations Theory model [20]. It appears that the “late majority” and “laggards”, approximately 50% of healthcare providers, will adopt the innovative entrusted budgets in Poland out of necessity, e.g., under pressure from patients and/or when there is no other option. Without the introduction of additional measures while maintaining the optionality of participation for service providers, it will be difficult to achieve the Ministry of Health’s goal of providing full coverage of the population with coordinated care in primary healthcare within the next 3–5 years [21]. This is especially the case since, over the next few years, the areas pointed out by service providers who do not deliver coordinated care (such as a lack of physicians and other staff, heavy workload, and additional new responsibilities) are unlikely to improve significantly. A similar situation occurred with the implementation of the electronic certificates of temporary incapacity for work in Poland, which is primarily issued by primary care physicians. Initially, paper sick leave certificates were still allowed, and the percentage of physicians who opted for electronic sick leave was very low. Therefore, after nearly three years, the requirement to issue sick leave certificates exclusively in electronic form was introduced, and the system has been in use since December 2018. It could be said that the universal electronic sick leave certificates was introduced at the last minute, as it proved very useful during the COVID-19 pandemic.

Implementing new payment models for health services in place of existing methods such as fee-for-service often improves the quality of care. The analysis of bundled-payment models around the world indicates that the introduction of new payment mechanisms improves the quality of care for most measured indicators, or alternatively has no significant impact on the quality of care [7]. In Poland, patients who have access to services rate coordinated care in primary healthcare within the entrusted budgets very positively. Primary healthcare facilities providing coordinated care ensure patients have access to diagnostic tests (e.g., ECG, echocardiography) in a much shorter time compared to waiting times in outpatient specialist clinics. For example, the waiting time for an ECG is on average 2.5 weeks versus 16 weeks outside of coordinated care [22].

The entrusted budgets were introduced in 2022 and were intended to be one of the ways to reduce the healthcare debt that arose due to the COVID-19 pandemic. The latest data from the Central Statistical Office (GUS) on the use of healthcare services by households indicate that there is a significant difference in the use of services currently compared to the period before the pandemic. In the fourth quarter of 2023, 29.1% of residents of Poland used healthcare services within primary healthcare, while in 2016, the corresponding figure was 35.9% (in 2020, this rate was 24.9%). Among those seeking advice in primary healthcare, the majority are individuals who visited a primary care physician once—45.8% of users. Individuals who should monitor their condition due to their health status do so relatively infrequently. In the fourth quarter of 2023, 47.0% of individuals with chronic diseases sought advice from a primary care physician. Additionally, 80.1% of performed imaging diagnostic tests were financed by NHF, while 19.9% were financed from sources outside the NHF in 2023. Even in the case of basic tests, such as blood counts, a large portion of tests (11.6%) are performed by patients outside the public health insurance system [23]. Primary healthcare services should be provided on the day of the request when justified by the health condition of the patient [14]. However, data from the Central Statistical Office indicate that the long waiting time for an advice session was the reason for 26.7% of individuals not seeking primary care physician advice, despite the need [23].

Based on previous research, it can be concluded that the impact of new methods of financing services on healthcare system expenditures is ambiguous [7,24]. It is currently difficult also to assess the impact of the introduction of the entrusted budgets to PHC providers in Poland on overall healthcare expenditures. These budgets increase expenditures in this part of the system, but are intended to reduce them in the long term in the areas of AOS and hospital care. The new financing mechanism is not yet fully formed and will most likely be subject to various modifications. The Ministry of Health plans to expand the scope of coordinated care to include the coordination of treatment of, among others, obesity and oncological support [21]. This is a good direction for change, but additional mechanisms should also be introduced to encourage all healthcare providers to offer these services that are already included in the entrusted budget. Otherwise, inequalities in access to primary healthcare services among patients will become even greater. Healthcare providers who are currently delivering coordinated care are likely to expand its scope, while healthcare providers who have not yet decided to offer coordinated care may still choose not to do so.

The analysis of the implementation of the coordinated care entrusted budget indicates that the financing model used for these services does not fully meet expectations. The healthcare provider should be interested in effectively treating the patient, rather than scheduling follow-up advice. This shows that changes are necessary and could include, for example, the implementation of mechanisms for paying for results, financial incentives for providers in exchange for effective treatment, the absence of complications, or ensuring that the patient does not end up in the AOS or hospital. A system of incentives for patients should also be introduced. The reward system could be linked, for example, to regular participation in health check-ups. The implemented solutions should be attractive enough for the patient to serve as an appropriate motivating factor for health-promoting behaviors.

A limitation of the analysis is that it includes contracts under which coordinated care services are provided; however, the service provider may not deliver all areas/fields of coordinated care. The service provider has the choice and can decide in which areas to provide services. Therefore, the estimated indicators also account for the ‘partial’ implementation of the coordinated care budget, directed only to some of the possible groups of patients with chronic disease.

Another limitation of the analysis is that it is based on data from February 2025, updated on 5 March. During this period, in several voivodships, the number of service providers who decided to sign a contract with the NHF and provide coordinated care increased by a few entities. It is likely that these numbers have increased again in the last few months. However, it should be noted that this applied only to a few voivodships, and the number of additional contracts was small compared to the overall number of contracts with the entrusted budget.

## 5. Conclusions

Access to coordinated care services within primary healthcare in Poland is currently uneven. Depending on the primary care physician a patient is treated by, they may have broad access to services or none at all. Considering the importance of these services for the population’s health capital, there is a clear need for further investments and to take actions that enhance both access to and utilization of these services. Primary health care in Poland is based on the principle of universality, and therefore access to all services, including those within the coordinated care entrusted budget, should be ensured for all patients, especially since preliminary research indicates that it improves the quality of care. Further long-term studies are necessary to assess whether access to services within the entrusted budget will improve in the coming years and to evaluate the effects of the implemented solution, both in terms of healthcare expenditure and quality of care. It should also be considered whether the assessed method of financing healthcare providers could be applied to other groups of healthcare services where patient participation is unsatisfactory and/or the motivation of service providers to provide services is lower, e.g., vaccinations, health checks and other preventive and health promotion programs.

## Figures and Tables

**Table 2:** Service providers delivering primary healthcare physician services, including those within the coordinated care entrusted budget in 2022–2025 at the voivodship level.

Voivodship	Number of Service Providers—Primary Healthcare Physician Services #				Number (%) of Service Providers with Services Within the Coordinated Care Entrusted Budget #				
	2022	2023	2024	2025	2022	2023	2024	2025	*p*-Value &
Dolnośląskie	495	485	470	464	18 (3.6%)	177 (36.5%)	209 (44.5%)	218 (47.0%)	0.1259
Kujawsko-Pomorskie	313	309	303	300	6 (1.9%)	108 (35.0%)	139 (45.9%)	141 (47.0%)	0.1134
Lubelskie	435	427	421	416	20 (4.6%)	247 (57.8%)	278 (66.0%)	276 (66.3%)	0.1702
Lubuskie	184	181	180	181	1 (0.5%)	37 (20.4%)	49 (27.2%)	53 (29.3%)	0.0835
Łódzkie	437	433	436	430	18 (4.1%)	153 (35.3%)	202 (46.3%)	231 (53.7%)	0.0584
Małopolskie	479	478	476	472	20 (4.2%)	164 (34.3%)	207 (43.5%)	219 (46.4%)	0.0964
Mazowieckie	701	695	694	685	32 (4.6%)	216 (31.1%)	305 (43.9%)	325 (47.4%)	0.0652
Opolskie	180	173	175	165	6 (3.3%)	24 (13.9%)	40 (22.9%)	41 (24.8%)	0.0509
Podkarpackie	338	345	332	322	2 (0.6%)	66 (19.1%)	106 (31.9%)	115 (35.7%)	0.0490
Podlaskie	249	247	243	242	4 (1.6%)	99 (40.1%)	117 (48.1%)	123 (50.8%)	0.1269
Pomorskie	303	304	296	295	14 (4.6%)	95 (31.3%)	117 (39.5%)	121 (41.0%)	0.1113
Śląskie	754	752	746	737	20 (2.7%)	193 (25.7%)	306 (41.0%)	317 (43.0%)	0.0601
Świętokrzyskie	185	182	183	180	3 (1.6%)	54 (29.7%)	83 (45.4%)	89 (49.4%)	0.0563
Warmińsko-Mazurskie	279	277	270	258	7 (2.5%)	51 (18.4%)	73 (27.0%)	73 (28.3%)	0.0871
Wielkopolskie	636	640	642	619	13 (2.0%)	106 (16.6%)	160 (24.9%)	174 (28.1%)	0.0501
Zachodniopomorskie	291	297	270	258	3 (1.0%)	50 (16.8%)	77 (28.5%)	80 (31.0%)	0.0657
Total	6259	6225	6137	6024	187 (3.0%)	1840 (29.6%)	2468 (40.2%)	2596 (43.1%)	0.0844

#—data as of 5 March 2025; &—*p*-value for linear trend (years 2022–2025); *p* > 0.05—not significant.

**Table 3:** Service providers delivering primary healthcare physician services, including those within the coordinated care entrusted budget in the counties of the Małopolskie Voivodship in 2025.

County	Number of Service Providers—Primary Healthcare Physician Services #	Number (%) of Service Providers with Services Within the Coordinated Care Entrusted Budget #
Bocheński	12	8 (66.7%)
Brzeski	13	6 (46.2%)
Chrzanowski	11	5 (45.5%)
Dąbrowski	9	2 (22.2%)
Gorlicki	19	9 (47.4%)
Krakowski	39	19 (48.7%)
Kraków	114	47 (41.2%)
Limanowski	23	13 (56.5%)
Miechowski	9	2 (22.2%)
Myślenicki	24	7 (29.2%)
Nowosądecki	38	20 (52.6%)
Nowotarski	26	14 (53.8%)
Nowy Sącz	8	5 (62.5%)
Olkuski	10	5 (50.0%)
Oświęcimski	13	6 (46.2%)
Proszowicki	10	1 (10.0%)
Suski	17	13 (76.5%)
Tarnowski	25	12 (48.0%)
Tarnów	12	7 (58.3%)
Tatrzański	9	4 (44.4%)
Wadowicki	17	8 (47.1%)
Wielicki	14	6 (42.9%)
Total	472	219 (46.4%)

#—data as of 5 March 2025.

## Data Availability

The datasets analysed during the current study are available in the NHF repository [https://baw.nfz.gov.pl/NFZ (accessed on 3 February 2025)] and NHF database [https://www.nfz.gov.pl/o-nfz/informator-o-zawartych-umowach/ (accessed on 5 March 2025)].

## Abbreviations

The following abbreviations are used in this manuscript:

PHC	Primary healthcare
NHF	National Health Fund (Narodowy Fundusz Zdrowia, NFZ)
AOS	Specialist outpatient care
GUS	Central Statistical Office

## References

[1] National Health Fund Informator o Terminach Leczenia [Information on Treatment Dates]. https://terminyleczenia.nfz.gov.pl/#.

[2] Watch Health Care Foundation Barometr WHC. http://www.korektorzdrowia.pl/barometr/.

[3] Mrożek-Gąsiorowska M., Tambor M. (2024). How COVID-19 has changed the utilization of different health care services in Poland. BMC Health Serv. Res..

[4] Waitzberg R., Gerkens S., Dimova A., Bryndová L., Vrangbæk K., Smith Jervelund S., Okkels Birk H., Rajan S., Habicht T., Tynkkynen L.-K. (2022). Balancing financial incentives during COVID-19: A comparison of provider payment adjustments across 20 countries. Health Policy.

[5] Ndayishimiye C., Tambor M., Behmane D., Dimova A., Dūdele A., Džakula A., Erasti B., Gaál P., Habicht T., Hroboň P. (2025). Health care provider payment schemes and their changes since 2010 across nine Central and Eastern European countries—A comparative analysis. Health Policy.

[6] Sagan A., Kowalska-Bobko I., Bryndová L., Smatana M., Chaklosh I., Gaál P. (2023). What is being done to respond to the rise of chronic diseases and multi-morbidity in Czechia, Hungary, Poland, and Slovakia?. Front. Public Health.

[7] Struijs J.N., De Vries E.F., Baan C.A., Van Gils P.F., Rosenthal M.B. (2020). Bundled-payment models around the world: How they work and what their impact has been. Commonw. Fund Issue Briefs.

[8] Ndayishimiye C., Tambor M., Dubas-Jakóbczyk K. (2023). Barriers and Facilitators to Health-Care Provider Payment Reform—A Scoping Literature Review. Risk Manag. Healthc. Policy.

[9] Hanson K., Brikci N., Erlangga D., Alebachew A., De Allegri M., Balabanova D., Blecher M., Cashin C., Esperato A., Hipgrave D. (2022). The Lancet Global Health Commission on financing primary health care: Putting people at the centre. Lancet Glob. Health.

[10] Sagan A., Kowalska-Bobko I., Badora-Musiał K., Gałązka-Sobotka M. (2022). A reform proposal from 2019 aims to improve coordination of health services in Poland by strengthening the role of the counties. Health Policy.

[11] National Health Fund Informator o Zawartych Umowach [Information on Signed Contracts]. https://www.nfz.gov.pl/o-nfz/informator-o-zawartych-umowach/.

[12] National Health Fund Baza Aktów Własnych NFZ [The Database of the Acts of the NHF]. https://baw.nfz.gov.pl/NFZ/tabBrowser/mainPage.

[13] National Health Fund Przychodnie POZ Realizujące Opiekę Koordynowaną [Primary Care Clinics Providing Coordinated Care]. https://koordynowana.nfz.gov.pl/przychodnie-poz-realizujace-opieke-koordynowana/.

[14] Rozporządzenie Ministra Zdrowia z Dnia 24 Września 2013 r. w Sprawie Świadczeń Gwarantowanych z Zakresu Podstawowej Opieki Zdrowotnej (Dz.U. 2013 poz. 1248, z późn. zm.) [Regulation of the Minister of Health of September 24, 2013, on Guaranteed Services in Primary Healthcare (Journal of Laws 2013, Item 1248, as Ammended)]. https://isap.sejm.gov.pl/isap.nsf/DocDetails.xsp?id=WDU20130001248.

[15] Rozporządzenie Ministra Zdrowia z Dnia 17 Czerwca 2022 r. Zmieniające Rozporządzenie w Sprawie Świadczeń Gwarantowanych z Zakresu Podstawowej Opieki Zdrowotnej (Dz.U. 2022 poz. 1293) [Regulation of the Minister of Health of June 17, 2022, Amending the Regulation on Guaranteed Services in Primary Healthcare (Journal of Laws 2022, Item 1293)]. https://isap.sejm.gov.pl/isap.nsf/DocDetails.xsp?id=WDU20220001293.

[16] Zarządzenie nr 79/2022/DSOZ Prezesa Narodowego Funduszu Zdrowia z Dnia 29 Czerwca 2022 r. w Sprawie Warunków Zawarcia i Realizacji Umów o Udzielanie Świadczeń Opieki Zdrowotnej w Rodzaju Podstawowa Opieka Zdrowotna, z Późn. zm. [Ordinance no. 79/2022/DSOZ of The President of the National Health Fund of June 29, 2022, Regarding the Conditions for Concluding and Implementing Contracts for the Provision of Healthcare Services in the Field of Primary Healthcare, as Amended]. https://baw.nfz.gov.pl/NFZ/document/43440/.

[17] Zarządzenie nr 124/2022/DSOZ Prezesa Narodowego Funduszu Zdrowia z Dnia 29 Września 2022 r. Zmieniające Zarządzenie w Sprawie Warunków Zawarcia i Realizacji Umów o Udzielanie Świadczeń Opieki Zdrowotnej w Rodzaju Podstawowa Opieka Zdrowotna [Ordinance No. 124/2022/DSOZ of the President of the National Health Fund of September 29, 2022, Amending the Regulation on the Conditions for Concluding and Implementing Contracts for the Provision of Healthcare Services in the Field of Primary Healthcare]. https://baw.nfz.gov.pl/NFZ/document/1476/.

[18] Zarządzenie nr 47/2025/DSOZ Prezesa Narodowego Funduszu Zdrowia z Dnia 30 Czerwca 2025 r. Zmieniające Zarządzenie w Sprawie Warunków Zawarcia i Realizacji Umów o Udzielanie Świadczeń Opieki Zdrowotnej w Rodzaju Podstawowa Opieka Zdrowotna [Ordinance No. 47/2025/DSOZ of the President of the National Health Fund of June 30, 2025, Amending the Regulation on the Conditions for Concluding and Implementing Contracts for the Provision of Healthcare Services in the Field of Primary Healthcare]. https://baw.nfz.gov.pl/NFZ/document/43575/.

[19] de Vries E.F., Drewes H.W., Struijs J.N., Heijink R., Baan C.A. (2019). Barriers to payment reform: Experiences from nine Dutch population health management sites. Health Policy.

[20] Rogers E.M. (2003). Diffusion of Innovations.

[21] Ministerstwo Zdrowia Zapowiada Szereg Zmian w Opiece Koordynowanej [The Ministry of Health Announces a Series of Changes in Coordinated Care]. https://www.prawo.pl/zdrowie/zmiany-w-opiece-koordynowanej-plany-resortu-zdrowia,529728.html.

[22] Fundacja My Pacjenci Opieka Koordynowana w POZ–Ocena Pacjentów–Wyniki Pierwszego Badania Opinii [Coordinated Care in Primary Healthcare–Patient Evaluation–Results of the First Opinion Survey]. https://mypacjenci.org/opieka-koordynowana-w-poz-ocena-pacjentow-wyniki-pierwszego-badania-opinii/.

[23] GUS Ochrona Zdrowia w Gospodarstwach Domowych w 2023 r. [Healthcare in Households in 2023]. https://stat.gov.pl/obszary-tematyczne/zdrowie/zdrowie/ochrona-zdrowia-w-gospodarstwach-domowych-w-2023-r-,2,8.html.

[24] Karimi M., Tsiachristas A., Looman W., Stokes J., Galen M.V., Rutten-van Mölken M. (2021). Bundled payments for chronic diseases increased health care expenditure in the Netherlands, especially for multimorbid patients. Health Policy.

